# A three arm cluster randomised controlled trial to test the effectiveness and cost-effectiveness of the SMART Work & Life intervention for reducing daily sitting time in office workers: study protocol

**DOI:** 10.1186/s12889-018-6017-1

**Published:** 2018-09-14

**Authors:** Charlotte L. Edwardson, Stuart J. H. Biddle, Alexandra Clarke-Cornwell, Stacy Clemes, Melanie J. Davies, David W. Dunstan, Helen Eborall, Malcolm H. Granat, Laura J. Gray, Genevieve N. Healy, Gerry Richardson, Thomas Yates, Fehmidah Munir

**Affiliations:** 1 Diabetes Research Centre, University of Leicester, Leicester, UK; 2 NIHR Leicester Biomedical Research Centre, Leicester, UK; 3 Institute for Resilient Regions, University of Southern Queensland, Springfield Central, QLD Australia; 4 School of Health Sciences, University of Salford, Manchester, UK; 5 School of Sport, Exercise and Health Sciences, Loughborough University, Leicestershire, UK; 6 Leicester Diabetes Centre, University Hospitals of Leicester, Leicester, UK; 7 Baker Heart and Diabetes Institute, Melbourne, VIC Australia; 8 School of Public Health, The University of Queensland, Brisbane, QLD Australia; 9 Department of Medicine, Monash University, Melbourne, VIC Australia; 10 Department of Epidemiology and Preventive Medicine, Monash University, Melbourne, VIC Australia; 11 School of Exercise and Nutrition Sciences, Deakin University, Burwood, VIC Australia; 12 School of Sport Science, Exercise and Health, The University of Western Australia, Perth, WA Australia; 13 Mary MacKillop Institute for Health Research, The Australian Catholic University, Melbourne, VIC Australia; 14 Department of Health Sciences, University of Leicester, Leicester, UK; 15 Faculty of Health Sciences, School of Physiotherapy, Curtin University, Perth, WA Australia; 16 Centre for Health Economics, University of York, York, UK

**Keywords:** Behaviour change, Sit-stand, Workplace, activPAL, Standing

## Abstract

**Background:**

Office-based workers typically spend 70–85% of working hours, and a large proportion of leisure time, sitting. High levels of sitting have been linked to poor health. There is a need for fully powered randomised controlled trials (RCTs) with long-term follow-up to test the effectiveness of interventions to reduce sitting. This paper describes the methodology of a three-arm cluster RCT designed to determine the effectiveness and cost-effectiveness of the SMART Work & Life intervention, delivered with and without a height-adjustable desk, for reducing daily sitting.

**Methods/design:**

A three-arm cluster RCT of 33 clusters (660 council workers) will be conducted in three areas in England (Leicester; Manchester; Liverpool). Office groups (clusters) will be randomised to the SMART Work & Life intervention delivered with (group 1) or without (group 2) a height-adjustable desk or a control group (group 3). SMART Work & Life includes organisational (e.g., management buy-in, provision/support for standing meetings), environmental (e.g., relocating waste bins, printers), and group/individual (education, action planning, goal setting, addressing barriers, coaching, self-monitoring, social support) level behaviour change strategies, with strategies driven by workplace champions. Baseline, 3, 12 and 24 month measures will be taken. Primary outcome: Objectively measured daily sitting time (activPAL3). Secondary outcomes: objectively measured sitting, standing, stepping, prolonged sitting and moderate-to-vigorous physical activity time and number of steps at work and daily; objectively measured sleep (wrist accelerometry). Adiposity, blood pressure, fasting glucose, glycated haemoglobin, cholesterol (total, HDL, LDL) and triglycerides will be assessed from capillary blood samples. Questionnaires will examine dietary intake, fatigue, musculoskeletal issues, job performance and satisfaction, work engagement, occupational and general fatigue, stress, presenteeism, anxiety and depression and sickness absence (organisational records). Quality of life and resources used (e.g. GP visits, outpatient attendances) will also be assessed. We will conduct a full process evaluation and cost-effectiveness analysis.

**Discussion:**

The results of this RCT will 1) help to understand how effective an important simple, yet relatively expensive environmental change is for reducing sitting, 2) provide evidence on changing behaviour across all waking hours, and 3) provide evidence for policy guidelines around population and workplace health and well-being.

**Trial registration:**

ISRCTN11618007. Registered on 21 January 2018.

## Background

Technological innovations and economic advances have led to increases in physical inactivity and sedentary behaviour [1]. Evidence indicates that it is not only important to be physically active for at least 150 min a week, but also to limit the number of waking hours spent being sedentary (i.e., sitting). A wealth of epidemiological evidence now exists that demonstrates that sedentary behaviour is associated with an increased risk of poor metabolic health [2] and chronic disease (type 2 diabetes, cardiovascular disease, some cancers and mortality) [3–6], often independently of BMI and physical activity.

Office workers are one of the most sedentary populations, with data showing that they spend 70–85% of time at work sitting, with over a third of total sitting time being accumulated in bouts of prolonged, unbroken sitting of 30 min or more [7]. Additionally, research has shown that workers who spend large proportions of their time sitting at work also spend more time sitting during leisure time than their less sedentary counterparts [8]. A small number of epidemiological studies using isotemporal substitution analysis have shown that substituting sitting time for standing and stepping was beneficially associated with markers of cardiometabolic health such as glucose, insulin, and inflammation [9–11]. Furthermore, a rapidly growing body of acute experimental evidence demonstrates that avoiding long bouts of sitting by incorporating short (e.g., 2–5 min) but frequent (e.g., every 20-30 min) bouts of more light intensity movement (standing and stepping) improves glucose, insulin, blood pressure and fatigue levels [12–18]. Such sitting reduction strategies have also been shown to reduce musculoskeletal (e.g., low back) discomfort in office workers [19].

Recent evidence suggests that high levels of moderate-to-vigorous physical activity (MVPA), for example at least 60–120 min per day, may offset the increased mortality risks associated with high levels of total sitting time (> 8 h/day) [20, 21]. However, the high levels of activity needed to be protective are unlikely to be achievable for most of the population. Indeed, a recent study of one million adults showed that 75% do not undertake this amount of MVPA [21]. Therefore, a first “behavioural” step could be to simply get people standing and moving more frequently as part of their day.

A review was published in 2016, and updated in 2018, summarising the effectiveness of workplace interventions for reducing sitting time at work [22, 23]. These interventions included physical workplace changes such as providing height adjustable desks to enable sitting or standing at work, pedalling workstations and treadmill desks, policy changes, information provision and counselling, and computer prompts. Providing height-adjustable workstations was the most frequently implemented intervention and was reported as the most promising for reducing sitting time at work (average reduction of 100 min/work day in the short term, 57 min/work day in the medium term) [23]. Whilst positive findings were observed, the review concluded that the quality of evidence was low to very low for most studies mainly because studies were poorly designed; for example, there was a lack of non-biased cluster randomised controlled trials, and generally small samples. They also concluded that ‘there is a need for larger cluster-randomised trials with longer term follow-up’.

To date only a handful of studies currently address these limitations [24, 25] and only one ongoing study, albeit that does not include a control group, has a follow up beyond 12 months [26]. The Stand More AT Work (SMArT Work) multi-component intervention was evaluated through a large cluster RCT with follow up at 3, 6 and 12 months [24, 27]. Although SMArT Work was successful in reducing sitting time at work et all follow up time points, results suggested that no changes in sitting outside of work were made as reductions seen for daily sitting time were similar to those observed during work hours [27]. Similarly, the Stand Up Victoria trial also observed significant changes in sitting time across the day, but findings suggested that this was also predominantly driven by workplace changes, rather than changes during non-work time [28]. To maximise the potential health benefits of reducing sitting time there is a need for interventions to take a ‘whole of day’ approach to behaviour change, and focus not only on workplace sitting time, but also on sitting outside of work.

In recent years, interventions focused on reducing sitting time in the workplace have involved providing the employee with a height-adjustable workstation to enable them to sit or stand to perform their work tasks. As mentioned previously, evidence indicates that this type of intervention shows promise for helping employees to reduce their sitting time [22, 23]. However, this piece of equipment is relatively expensive. Moreover, while the recent systematic review of workplace interventions suggests that greater reductions in sitting are seen with interventions involving height-adjustable workstations in comparison to interventions that do not (e.g., information provision and counselling), a direct comparison of a sitting reduction intervention with and without the provision of a height-adjustable workstation has not been conducted within a single, large, robustly designed study. This is an important next step as it will allow researchers and employers to understand the importance of providing a simple, but relatively expensive environmental change for significant reductions in sitting. One active research trial in the US is evaluating the efficacy of a multilevel intervention with (STAND +) and without (MOVE +) the provision of sit-stand workstations and although a large randomised trial, it does not include a control group [26].

### Aims and objectives of the study

#### Aim

The aim of this study is to determine the long-term effectiveness and cost-effectiveness of the multi-component SMART Work & Life intervention (when provided with and without a height-adjustable workstation) for reducing daily sitting time in office workers compared with no intervention. If both interventions are shown to be effective, a secondary aim will be to determine if one intervention is more effective than the other. SMART Work & Life is a refined and extended version of the previously evaluated SMArT Work intervention [24, 27].

#### Primary objective

To investigate the impact of SMART Work & Life, delivered with and without a height adjustable workstation, on objectively measured daily sitting time compared to usual practice at 24-months follow-up.

#### Secondary objectives

To investigate the impact of SMART Work & Life, delivered with and without a height adjustable workstation, over the short (assessed at 3 months), medium (assessed at 12 months) and longer term (assessed at 24 months) on;

- daily sitting time (3 and 12 months)
- sitting time during working hours
- daily prolonged sitting time and inside/outside of working hours
- daily standing time and inside/outside of working hours
- daily light and moderate-to-vigorous physical activity and inside/outside of working hours
- daily stepping time and number of steps and inside/outside of working hours
- adiposity (BMI, percent body fat, waist circumference)
- blood pressure
- blood markers (e.g. blood glucose, cholesterol, triglycerides)
- musculoskeletal issues
- psychosocial variables (e.g. fatigue, stress, anxiety and depression, work engagement, job performance and satisfaction, presenteeism, sickness absence, and quality of life)
- sleep

We will also conduct a full process evaluation and a full economic evaluation.

## Methods

### Design and randomisation

**Fig. 1:**
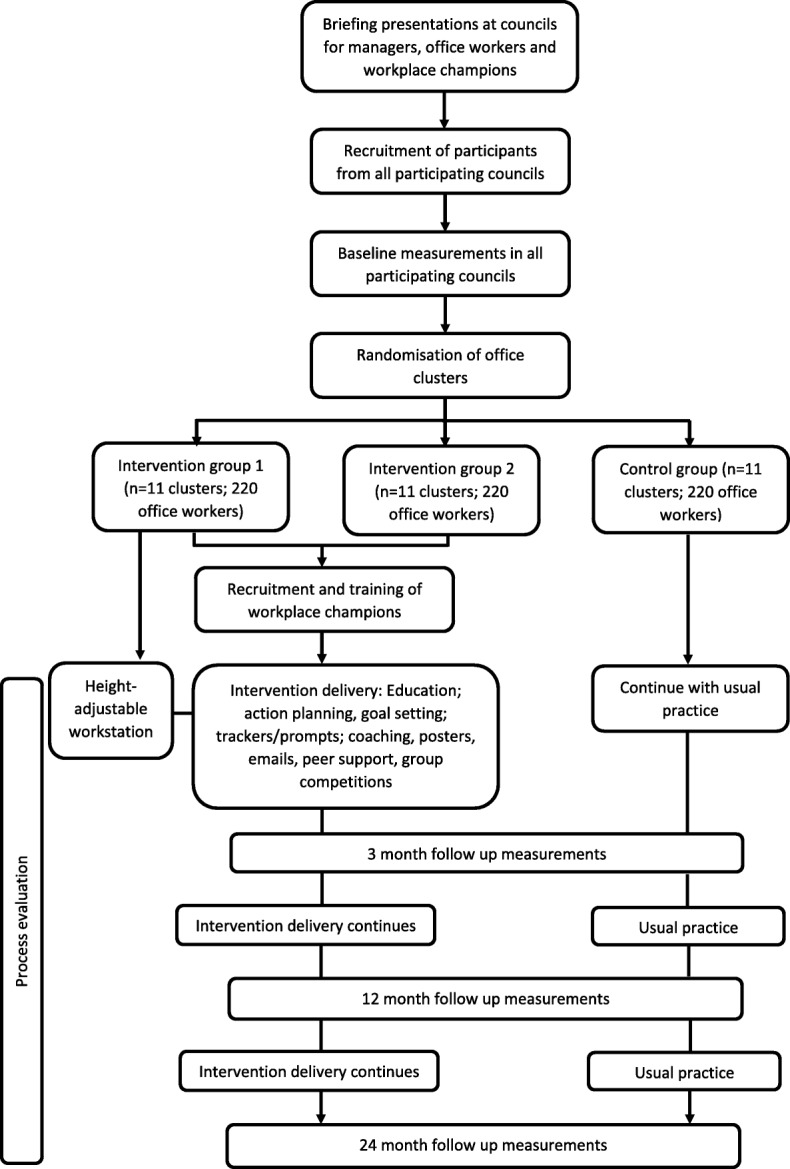
Study design

This is a three-arm cluster randomised controlled trial (RCT) aiming to recruit 33 clusters. Clusters will be randomised at the office level to reduce the risk of contamination. Using computer generated lists, office groups (clusters) will be randomised by a statistician from the Leicester Clinical Trials Unit to one of the three groups, stratified by Council area (e.g., Leicester) and cluster size (< 10 participants, ≥10 participants). Randomisation will be performed in batches after clusters have completed their baseline measures. The clusters will be recruited from Councils within the Leicester, Manchester and Liverpool areas in the UK. We aim to recruit 660 office workers across the 33 clusters. Clusters will be randomised to receive one of the following conditions: 1) The multi-component SMART Work & Life intervention with a height-adjustable workstation (intervention 1), or 2) The multi-component SMART Work & Life intervention without a height-adjustable workstation (intervention 2) or 3) usual practice (control condition). Measurements will be repeated, using identical standardised procedures, at 3 months to assess any short-term changes and 12 months and 24-months to assess any longer term changes. Observations, questionnaires and focus groups with office workers and workplace champions will be conducted throughout the intervention period as part of our full process evaluation. This study will be conducted, analysed and reported according to the Consolidation Standards of Reporting Trials (CONSORT) statement for cluster RCTs. Given the nature of the study it is not possible to blind the participants to which intervention they receive. Measurement team members, apart from the team lead, were blinded to group randomisation. Ethical approval has been sought and obtained from the University of Leicester’s College of Life Sciences and University of Salford’s Research, Enterprise and Engagement Ethical Approval Panel. The University of Leicester will act as study sponsor. Figure 1 shows the overall study design.

### Setting

This study is targeting office workers within City and Borough Councils within the Leicester, Manchester and Liverpool areas, UK.

### Cluster and participant recruitment

The study will be advertised in several ways (these have been informed by Councils themselves):

- Using the councils’ weekly newsletter
- Using the councils’ intranet
- Through Manager meetings
- Displaying posters on display boards
- Through more targeted strategies directly to appropriate office-based departments e.g., office walk arounds

It will be made clear in the study adverts that the study is looking to recruit office-based employees who spend the majority of their work and waking day sitting down (see inclusion criteria) and that the study will be looking to recruit as many employees within the same office space as possible. Employees who want to hear more about the study will be invited to a presentation where the study will be explained. At the end of the presentation participant information sheets and reply slips (which will include a pre-screening questionnaire to gather basic information to assess eligibility) will be given out to employees who are interested in taking part. The study team’s contact details will also be provided on all study recruitment material so that interested employees can request a participant information sheet directly from the research team.

### Inclusion and exclusion criteria

Participants who meet the following criteria will be eligible:

- Spend the majority of their day sitting (> 50%). This will initially be self-reported and be used as screening criteria prior to the consent and baseline measurement visit. This will subsequently be confirmed using the objective data collected via the activPAL device (see measures section).
- Work for the council at least 60% full time equivalent.
- Work in the same office at least 3 days per week.
- Be willing and able to give informed consent to take part in the study.
- Able to walk without the use of an assistive device or requiring assistance from another person.

The participant may not enter the study if any of the following apply:

- They are currently pregnant.
- Currently using a height-adjustable workstation at their primary work location.
- Unable to communicate in English.
- Unable to provide written informed consent.

### Sample size

The primary analysis will be performed using linear multilevel models, which require a minimum of 10 clusters per arm in order to robustly estimate random effects [29]. Power calculations indicated that with a sample size of 10 clusters per arm and an average cluster size of 14, this study would have over 90% power to detect a 60 min reduction in overall sitting time with a two-tailed significance level of 5%. The calculations assumed a standard deviation of 90 min (informed by SMArT Work, [27]), a conservative intraclass correlation coefficient (ICC) of 0.05 (informed by Stand Up Victoria, [28]), a coefficient of variation to allow for variation in cluster size of 0.54. These calculations allowed for multiple comparisons against the control group. The number of clusters per arm has been inflated by 1 to allow for whole cluster drop out and the sample size was also inflated by 30% to allow for potential individual loss to follow-up and non-compliance with the primary outcome (inflating the n per cluster from 14 to 20). This gives a total sample size of 660 to be recruited, with 11 clusters per arm. Finally, the sensitivity of power was assessed against alternative ICC values of 0.021 and 0.10 [28]. Adequate power for RCTs is widely accepted as 80%, and with these ICC values the power was above the required level at 98 and 81%, respectively.

### Intervention conditions

#### Background

The SMART Work & Life intervention is a multicomponent intervention promoting positive changes in daily sitting and movement in office workers. SMART Work & Life has been developed with input from office workers, local council office workers, workplace champions, council stakeholders, recently published research, experiences in Australia (the Stand Up Australia programme of research e.g., Stand Up Comcare and Stand Up Victoria) [30], and a 12-month RCT of a previous version of the intervention - SMArT Work [24, 27, 31]. As a result, SMArT Work has been refined and extended to become SMART Work & Life. SMART Work & Life incorporates improvements that were noted following the SMArT Work RCT and addresses the gaps in existing interventions by going beyond sitting in the workplace to also focus on behaviour change outside of work, emphasising a novel ‘whole-of-day’ preventive approach to sitting reduction.

SMART Work & Life is grounded in several behaviour change theories (Social Cognitive Theory [32], Organisational Development Theory [33], Habit Theory [34], Self-Regulation Theory [35] and Relapse prevention Theory [36]) and implemented through the Behaviour Change Wheel (BCW) and the associated COM-B approach [37].The latter has ‘capability’, ‘opportunity’, and ‘motivation’ as central components in guiding ‘behaviour’.

#### Intervention goal

The aim of the intervention will be to promote and maintain at least a 60 min per day reduction in daily sitting time compared to usual practice at 24 months. Recent experimental evidence has demonstrated a reduction in glucose, insulin and blood pressure following regular standing and walking breaks (i.e., every 20–30 min), with total reductions in sitting varying from 28 min to 60 min across the course of a day [12, 13, 15, 16, 18]. Furthermore, using statistical modelling we have observed that interchanging 30 mins/day of sitting (measured with the activPAL) with standing and stepping is associated with favourable differences in insulin sensitivity [9] and inflammation [10]. In a similar analysis, interchanging 2 h of sitting/day with standing or stepping was associated with favourable differences in glucose, triglycerides, cholesterol and waist circumference [11]. Others have also shown that each additional hour/day of sitting past 7 h and 8 h is associated with a 5 and 4% higher risk of all-cause mortality in the general population [38, 39]. Thus based on the available evidence, a reduction in sitting time of 60 min per day is likely to represent a clinically meaningful difference in behaviour.

#### Main intervention components

*Organisational strategies* grounded in Social Cognitive Theory and Organisational Development Theory (targeting ‘opportunity’ & ‘motivation’ through BCW intervention functions: enablement, persuasion, environmental restructuring, modelling, positive emotion): 1) we will seek buy-in from the management through the briefing events by explaining the importance of reducing and breaking up sitting at work and how this may lead to workplace benefits without negatively affecting performance and productivity; 2) a brief awareness session (online/video) will reinforce the benefits for the workforce and employers of reducing sitting time in and outside of work and encourage them to brainstorm organisational strategies that could take place, review any current policies around being active at work as well as create new policies around topics such as standing and walking meetings, provision for lunch time walking, internal competitions and displaying signs around the workplace. We will also encourage managers to review the layout of their office space to promote increased movement of staff e.g., location of printers, waste bins, water coolers; 3) Modelling of the positive behaviour from managers will also be emphasised.

*Environmental strategies* grounded in Social Cognitive Theory, Organisational Development Theory and Habit Theory (targeting ‘capability’, ‘motivation’ & ‘opportunity’ through BCW intervention functions: environmental restructuring, enablement as well as ‘automatic’ forms of motivation, including emotion): 1) Small-scale environmental restructuring in the office and at home (e.g., relocation of printers and waste bins), 2) Motivational and reminder signs around the office space and at home to sit less and move more, 3) A height-adjustable workstation to allow the individual to sit or stand to work. The individual will get a choice of desk platform within a set budget. This allows flexibility for office set up, participant preference and avoids testing the effectiveness of a specific type of desk rather than the concept.

*Individual and group strategies* grounded in Social Cognitive Theory, Self-Regulation Theory and Relapse Prevention Theory (targeting ‘capability, ‘motivation’ & ‘opportunity’ through BCW intervention functions: enablement, persuasion, education and training): 1) An initial online education session which covers health consequences of sitting and benefits of reducing and regularly breaking up sitting. The session encourages participants to estimate their own sitting time, brainstorm strategies to reduce sitting at work and outside of work, consider barriers to reducing and breaking up sitting and ways to overcome these barriers. At the end of the session individuals will be encouraged to set a goal around sitting less and an action plan to achieve this. The focus on overall daily sitting will be emphasised rather than just workplace sitting; 2) Self-monitoring of sitting behaviour across the whole waking day will be encouraged through the use of free computer prompts, timers and mobile phone apps. For example, the Rise & Recharge app. The importance of self-monitoring and prompts will be introduced during the education session and the choice of apps, computer software and prompts will be presented during the session; 3) Workplace champions will receive training to encourage their colleagues, deliver brief group coaching, implement competitions and send emails. The coaching sessions will be used to discuss progress, review goals and action plans, discuss personal or social barriers and any benefits experienced. These coaching sessions will take place four times during the intervention period; 4) Workplace champions will send out monthly motivational/education emails; 5) Social support, from colleagues and family members, will be encouraged through regular activity competitions inside and outside of work.

#### Intervention arms 1 and 2

Both intervention arms will receive all of the intervention components listed in the previous sections. However, intervention arm 2 will not receive a height-adjustable workstation. By having both intervention arms receive the same intervention with the exception of the workstation will allow us to investigate how important providing this component is for reducing sitting.

#### Control arm

Office clusters assigned to the usual practice control arm will be asked to continue with their usual occupational health promotion conditions. Participants in the control arm will be asked to complete the same study measurements as those in the intervention arms, at the same time points.

### Measurements

The outcome measurements (unless stated otherwise) will be assessed at four time points; baseline and 3, 12 and 24 months following baseline by a team of researchers who have undergone relevant training. At the baseline visit, the study will be fully explained to the participant again and written informed consent will be obtained if the participant is happy to take part in the study. The consent procedure will be performed by a researcher with appropriate consent training. Only once informed consent has been given will measurements be taken.

#### Objectively measured sitting and physical activity

The activPAL™ micro device will be worn on the thigh 24 h/day for 7 days. It will be made waterproof using a nitrile sleeve and waterproof medical dressing. This device will assess a variety of aspects of behaviour including sitting, standing and stepping time (total and light and moderate-to-vigorous), prolonged sitting and standing, number of steps and number of transitions from sitting to an upright posture. These variables will be calculated daily (i.e., across all waking hours) and during work hours only. Participants who provide an adequate number of valid days will receive a £10 voucher at the end of each data collection time point. The activPAL is commonly used in sedentary behaviour and physical activity research [40] and has been found to be a valid and reliable measure of sitting, standing, stepping and postural transitions in adults [41–43]. The activPAL data will be cleaned and processed using a previously published automated algorithm [44].

A wrist-worn accelerometer will also be worn on the non-dominant wrist 24 h/day for 7 days. Time spent in different intensities of physical activity as well as sleep duration and other sleep variables such as efficiency will be calculated.

Participants will be asked to complete a short log each day to note the time they went to bed, went to sleep, woke up and got out of bed each day, work times, as well as recording any periods throughout the day if they removed the devices.

#### Self-reported lifestyle behaviours

Participants will be asked to complete an adapted version of the Occupational Sitting and Physical Activity Questionnaire [45], an adapted version of the past day recall of sedentary time questionnaire, which asks about sitting outside of work in certain contexts [46], as well as estimate the hours they spend sitting and breaking up sitting as part of their job [47]. Participants will be asked to estimate the percentage of their working day that they spend at their desk space and their office space.

Dietary behaviours and alcohol intake will be assessed using questions from the Whitehall II study [48]. Information on smoking status (current smoker, past smoker, non-smoker) will also be gathered by self-report.

Self-report sleep duration and quality will be assessed using the Pittsburgh Sleep Quality Index [49].

#### Demographic, medical history and medication

During their baseline visit, participants will be asked their age and date of birth, ethnicity, education level, current job role and pay grade, working site, working hours, length of time in post, number of people in their office and department, postcode and household composition. At each follow up visit, participants will be asked if there has been any change in these aspects.

Details of any history of disease or injuries that may indicate an inability to participate in the study will be measured. If needed, results will be reviewed to define eligibility. Medication will also be recorded.

#### Anthropometrics and blood pressure

Height will be measured in centimetres (cm), to 1 decimal place, using a Leicester portable height measure. Waist circumference will be measured using a standard anthropometric tape measure, with the tape measure being placed around the abdomen midway between the uppermost border of the iliac crest and the lower edge of the chest (thorax) formed by the bottom edge of the rib cage. A reading in cm, to 1 decimal point, will be taken when the tape is snug, but not compressing the skin. Weight, in kilograms (kg), and body composition will be measured using a Body Composition Analyser. Participants will remove shoes, socks and heavy outerwear clothing and ensure their pockets are empty before stepping on to the scales. Body mass index (BMI) is calculated by the scales as kg/m^2^. Blood pressure (BP) will be assessed using an Omron automated blood pressure monitor (Omron Healthcare Europe). Participants will be asked to sit quietly and relax prior to having their BP measurements taken and three readings will be taken, with the average of the last two readings used in the analyses.

#### Biochemical assessments

The Quo-Test® HbA1c Analyser (point-of-care device; EKF Diagnostics, Cardiff, UK) will be used to measure glycated haemoglobin. Additionally, we will use the Cardiochek® PLUS point-of-care analyser (PTS Diagnostics, IN, USA) to measure circulating cholesterol (total, HDL, LDL), triglycerides and glucose. Capillary blood samples will be taken from each participant using the finger prick method. The CardioChek® PLUS system, which is a portable hand-held device that requires between 15 and 40 μL (millions per microliter) of blood taken using a finger-stick, will be used for these measurements. No blood will be stored and all blood contaminated testing sticks will be deposed of appropriately. Participants will be asked to fast (no food or drink except water) for at least 10 h prior to the blood tests. All participants will receive feedback on these results.

#### Work-related measures

Job performance [50] and job satisfaction [51] will be measured using single-item 7-point likert scales. Work engagement (characterized by vigour, dedication, and absorption) will be measured using the Utrecht Work Engagement Scale [52]; a multi-item 7-point likert scale. The Need for Recovery Scale [53] will be used to measure occupational fatigue. Musculoskeletal symptoms will be assessed using the Standardised Nordic Questionnaire [54]. Work load and relations will be assessed using the demands, control and support scales from the Health and Safety Executive Management Standards Indicator Tool using a 5-point likert scale [55]. Sickness presenteeism will be assessed by the validated 8-item Work Limitations Questionnaire [56] which asks participants to rate on a six-point Likert scale how their health has affected aspects of their work in the past 2 weeks. Data on sickness absence will be collected using both self-report and from employer records and include frequency and duration of self-certified and certified sickness. Reasons for sickness absence will also be recorded. Data on sickness absence will be collected for 12 months prior to the intervention and for the 24 months of the intervention period.

#### Social norms, cohesion, support and strategies for sitting less

Organisational social norms will be assessed using eight items (e.g., ‘My workplace is committed to supporting staff choices to stand or move more at work’) on a 5-point Likert [25]. To capture the presence and extent of cohesion, cooperation and community in workplace teams the ‘social community’ sub-scale of the Copenhagen Psychosocial Questionnaire-II [57] will be used. This sub-scale uses three 6-point Likert scale items. Participants will be asked about the support they have received from the organisation, manager, colleagues and family for sitting less and moving more often [58]. Participants will be asked to report the frequency of any strategies they have used to sit less and move more often [58].

#### Mental health, well-being and quality of life

Health-related quality of life will be assessed using the EQ5D-5 L [59]. Anxiety and depression will be measured using the Hospital Anxiety and Depression Scale [60]. Stress will be measured using the Perceived Stress Scale [61]. Emotion will be assed using the Positive and Negative Affect Schedule which comprises two mood scales (positive and negative) [62]. Wellbeing will be measured using the World Health Organisation-Five Well-Being Index (WHO-5) [63].

#### Physical and mental fatigue

The Fatigue Scale [64] will be used to measure fatigue severity. The Fatigue Scale is one of the most widely used measures assessing fatigue and includes 11 items, seven assessing physical fatigue and four assessing mental fatigue. Responses to items are measured using a 4-point Likert-scale.

#### Health-related resource use

The health-related resource use will be based on a variant of the Client Service Receipt Inventory [65] and will include services that this population are likely to utilise such as GPs and Practise nurse appointments, occupational health visitors and other professionals that are deemed appropriate.

#### Workplace and workplace champion characteristics

At baseline each cluster will be asked to complete a short audit of their work environment. Basic information about each workplace champion in the intervention clusters will be collected at baseline e.g., gender, age, job role, length of experience being a workplace champion.

#### Process evaluation

The process evaluation methods will be a mix of questionnaires, interviews, focus groups and direct observation. The process evaluation will be used to understand: the participants’ experiences of the intervention and its different components; any discrepancies between expected and observed outcomes; the influence of intervention components and context on the observed outcomes; sustainability; the extent of any contamination between intervention and control; and, any unexpected events arising from participation. Completion of education and attendance at coaching sessions will be recorded. Self-report questionnaires provided to study participants will evaluate their opinions of the various intervention components (e.g. education, coaching, self-monitoring, workstation). Interviews and focus groups with study participants (sub-sample) will further examine engagement in the various components of the intervention, along with any barriers or facilitators to participating in the various components. Focus groups with workplace champions will further examine the intervention implementation and the champions’ experiences of delivery. All interviews and focus groups will be audio-recorded and transcribed verbatim.

Throughout the intervention we will monitor the fidelity of the intervention implementation using the Normalisation Process Theory framework [66] in line with guidance from the National Institutes of Health Behaviour Change Consortium and the DESMOND collaborative. Observations or recordings (via voice recorder) of sessions (e.g., coaching) will take place in both intervention arms to assess whether the content was delivered as expected and receipt of the intervention by attendees. During the observations a case report form will also be completed. The case report form will combine an ‘adherence measure’ to capture delivery (mode of delivery (dose)/duration/content) and use of resources (materials/activities). The structured observation tool will assess facilitator delivery of prescribed behaviours and behaviour change techniques. The case report form will also contain specific objective ‘receipt’ measures and will likely include examples related to how well the participants understand the content and engage in the session.

Observations in the office clusters will also take place in a random sample of offices in both intervention arms at several time points during the intervention period. Each observation will be performed over one whole working day. This observation work will be guided by the four domains of the Normalisation Process Theory and an observation guide will highlight the types of behaviours of focus, such as: use of height adjustable workstation, sitting and standing time, engagement with colleagues, walking/standing meetings as well as office structure, posters displayed. Practically, the observation work will include keeping structured field notes and collating relevant documentation for further context and insight, and may include informal discussions with office workers and workplace champions. A random sample of control offices will also be observed to judge contamination and other practices that may impact on our behaviours of interest.

### Statistical analysis

#### Primary and secondary outcomes

The aim of the primary analysis is to investigate the impact of the multi-component intervention (SMART Work & Life), with and without a height-adjustable workstation, on objectively measured daily sitting time compared to usual practice at 24-month follow-up. The primary outcome analysis is powered to detect a significant difference in sitting time of 60 min/day at 24 months. However, discontinuation of the study due to futility will be considered in a formal interim analysis at 12 months. An Independent Data Monitoring Committee (DMC) will be convened to review the primary outcome at 12 months. The conditional probability of the final study results being statistically significant given the data observed at 12 months will be calculated and the DMC will make a recommendation based on this and other important factors (i.e. trial conduct, data quality, participant retention) of whether or not to continue follow-up until 24 months. If the DMC decide the data from the interim analysis at 12 months provides satisfactory evidence to continue, the trial will continue to follow-up participants to 24 months. Furthermore, only if both arms are determined to be futile at the interim analysis stage will the trial be stopped early.

The primary analysis will be performed using a linear multilevel model with sitting time as the outcome variable. Levels to indicate the clustering of workers within office sites, a categorical variable for randomisation group as the explanatory variable (control will be the reference group and will be compared to intervention 1 and intervention 2), and terms for the stratification factors (area and cluster size), baseline values and device wear time will be confounders. In these linear multilevel models, office clusters will be incorporated as a random effect to model worker heterogeneity within office sites. The structure of the variance-covariance matrix for the random effect will be assumed to be unstructured and the models will be estimated using restricted maximum likelihood. For the primary analysis, missing data will not be replaced (complete case analysis) and participants will be included in the intervention group in which their clusters were randomised irrespective of the intervention that was actually received.

The baseline characteristics of those who have complete primary outcome data will be compared with those who dropped out from the study in order to investigate differences between them.

A sensitivity analysis using multiple imputation will be performed to evaluate the impact of missing outcome data on the results obtained and to account for uncertainty associated with imputing data (full intention-to-treat analysis). Missing data will be replaced using multiple imputation methods in Stata using the MI command. The effect size will also be estimated using a per-protocol analysis, which will only include those who were compliant with the protocol and follow-up visits. Secondary outcomes, including those measured at other time-points, will be analysed using similar methodology. We will additionally assess data from all time points for the primary outcome in a single analysis using repeated measured. We will also conduct a subgroup analysis which compares the treatment effect in those clusters in which other work place health initiatives were taking place at the same time as the study compared to those where there were no such initiatives.

All tests and reported *p*-values will be two-sided. Estimates will be presented with 95% confidence intervals.

#### Process evaluation

Questionnaire data will be summarised using frequency counts and means (± SD) where appropriate. Audio-recordings of interviews and focus groups with office workers and workplace champions will be transcribed verbatim and analysed using framework analysis using the Normalisation Process Theory as the overarching framework.

#### Cost effectiveness

The economic evaluation will consist of two analyses. i) a cost-consequence analysis based on the observed results within the trial period and ii) a cost-effectiveness analysis where differences between groups in the trial will be extrapolated to the longer term where appropriate.

For both analyses, costs in both arms will be estimated from a National Health Service (NHS) and Personal Social Services (PSS) perspective (consistent with that used by the National Institute for Health and Care Excellence (NICE)) as well as a wider public sector perspective. In each analysis, the cost of the SMART Work & Life groups will include an estimate of the cost of the intervention, with and without the height adjustable desk. The cost of the intervention consists of the cost of equipment (such as desks) and the cost of training and delivery of intervention. We will estimate the cost of the equipment from manufacturer’s estimates of costs. We will also estimate the cost of training and delivery of intervention by the workplace champions.

##### Within-trial analysis

Within the period of the trial, we will collect resource use estimates from participant questionnaires. These questionnaires will record health related resource use as well as absence from employment. The health related resource use will be based on a variant of the Client Service Receipt Inventory and will include services that this population are likely to utilise such as GPs and Practice nurse appointments, occupational health visitors and other professionals that are deemed appropriate. Costs of resources will be calculated by applying published national unit cost estimates (e.g. NHS reference costs or Personal Social Services Research Unit costs of health and social care), where available, to estimates of relevant resource use.

A range of outcomes will be assessed in the trial including health related quality of life, measured using the EQ5D-5 L The within trial analysis will present incremental results for the primary and secondary outcomes (including EQ5D) in both intervention and control arms and will be compared with the incremental costs measured above. We will also present the results in terms of the differences in time absent from work between the groups. As there is some controversy over inclusion of productivity losses in the assessment of cost-effectiveness, the within trial analysis will be presented both with and without estimates of the cost of sickness absenteeism. This will allow decision makers to assess the importance of inclusion of absenteeism costs when deciding whether to implement the intervention.

##### Longer term analysis

While there may be short term health benefits from reducing levels of sitting time, the longer term effects on mortality on office workers is likely to be more important. We will therefore use existing evidence that links short-term trial endpoints and longer term outcomes. While some existing evidence used covariates to adjust for confounding factors, it is not possible to assess unmeasured confounders. Therefore, we will use existing evidence to extrapolate costs and effects to a more appropriate time horizon; however, as recommended by Taylor and Elston [67] we will explain how the surrogate-final outcomes relationship is quantified and explore the uncertainty around the use of the surrogate outcome (in this case sitting time) through sensitivity analysis.

If appropriate an Incremental Cost-effectiveness Ratio for the extrapolated period will be reported using the Quality Adjusted Life Year (QALY). As with the within-trial analysis, we will conduct analyses where productivity losses are included/excluded to assess the impact on decision making. Costs and effects will be discounted at the prevailing recommended rate (currently 1.5% per annum on both costs and effects), but will be the subject of sensitivity analysis to reflect the ongoing uncertainty around appropriate discount rates for public health interventions. We will conduct probabilistic sensitivity analyses to allow a characterisation of the uncertainty around the adoption decision which we will depict using cost-effectiveness acceptability curves. Sensitivity analyses will be performed to determine the robustness of the results to altering certain assumptions such as the discount rate, inclusion/exclusion of productivity losses and the robustness of the relationship between sitting time and mortality.

### Data handling

All data collected will be kept strictly confidential and in accordance with all relevant legislation. The participants will be identified on documentation by a unique ID number, not by name (apart from on the consent form and enrolment log). All research data will be kept in a secure location within University of Leicester, University of Salford or the University Hospitals of Leicester, accessible only by named members of the research team during the active phase of the study and until the data have been analysed. It will then be archived in line with University of Leicester policy. The Leicester Clinical Trials Unit will be providing a Good Clinical Practice compliant database solution using a Clinical Data Management System called InferMed Macro. This is a secure and validated database solution with quality control mechanisms to ensure that the data collected are complete and accurate.

## Discussion

Office workers exhibit high levels of sitting both inside and outside of work [7, 8], with accumulating evidence demonstrating the detrimental impacts of high levels of sedentary behaviour on health [3–6] interventions in this population are needed. Although previous workplace interventions have shown promise in reducing sitting time [22, 23] study designs have been of low quality [22, 23], with only a handful of fully powered cluster randomised controlled trials being conducted [24, 25].

Strengths of this study include the robust randomised controlled trial design, with randomisation at the cluster level to reduce contamination, the large fully powered sample, the inclusion of short, medium and longer term follow up measures, the extensive quantitative and qualitative process evaluation, the rigorous economic evaluation and the use of an objective measure of physical activity as the primary outcome. Furthermore, this study is unique, in that it will be delivered at two levels i.e., with and without the provision of a height-adjustable workstation. These study aspects will all contribute to building the evidence base around the effectiveness of interventions to reduce sitting time and inform policy guidelines for population and workplace health and wellbeing.

## Abbreviations

BCW: Behaviour change wheel
BMI: Body mass index
BP: Blood pressure
COM-B: Capability, opportunity, motivation-behaviour
CONSORT: Consolidation Standards of Reporting Trials
DMC: Data monitoring committee
GP: General practitioner
HDL: High-density lipoprotein
ICC: Intraclass correlation coefficient
LDL: Low-density lipoprotein
MVPA: Moderate-to-vigorous physical activity
NHS: National Health Service
NICE: National Institute for Health and Care Excellence
PSS: Personal social services
QALY: Quality adjusted life year
RCT: Randomised controlled trial
WHO-5: World Health Organisation-Five Well-Being Index
